# Smart CAR-T Nanosymbionts: archetypes and proto-models

**DOI:** 10.3389/fimmu.2025.1635159

**Published:** 2025-08-12

**Authors:** Juan C. Baena, Juan Sebastián Victoria, Alejandro Toro-Pedroza, Cristian C. Aragón, Joshua Ortiz-Guzman, Juan Esteban Garcia-Robledo, David Torres, Lady J. Rios-Serna, Ludwig Albornoz, Joaquin D. Rosales, Carlos A. Cañas, Gustavo Adolfo Cruz-Suarez, Felipe Ocampo Osorio, Tania Fleitas, Ivan Laponogov, Alexandre Loukanov, Kirill Veselkov

**Affiliations:** ^1^ Division of Oncology, Department of Medicine, Fundación Valle del Lili, ICESI University, Cali, Colombia; ^2^ Division of Hematology, Department of Medicine, Fundación Valle del Lili, ICESI University, Cali, Colombia; ^3^ Division of Rheumatology, Department of Medicine, Fundación Valle del Lili, ICESI University, Cali, Colombia; ^4^ Universidad Icesi, CIRAT: Centro de Investigación en Reumatología, Autoinmunidad y Medicina Traslacional, Cali, Colombia; ^5^ Artificial Intelligence Unit, Fundación Valle del Lili, Cali, Colombia; ^6^ LiliCAR-T Group, Fundación Valle del Lili, ICESI, Cali, Colombia; ^7^ Director of Clinical Research, Rio Grande Urology, El Paso, TX, United States; ^8^ Departamento de Salud Pública y Medicina Comunitaria, Universidad ICESI, Cali, Valle del Cauca, Colombia; ^9^ Centro de Investigaciones Clínicas, Fundación Valle del Lili, Cali, Valle del Cauca, Colombia; ^10^ Department of Chemistry and Materials Science, National Institute of Technology, Gunma College, Maebashi, Japan; ^11^ Laboratory of Engineering Nanobiotechnology, University of Mining and Geology "St. Ivan Rilski", Sofia, Bulgaria; ^12^ Department of Medical Oncology, Hospital Clínico Universitario, INCLIVA, Biomedical Research Institute, University of Valencia, Valencia, Spain; ^13^ Division of Cancer, Department of Surgery and Cancer, Faculty of Medicine, Imperial College London, London, United Kingdom; ^14^ Department of Environmental Health Sciences, Yale School of Public Health, New Haven, CT, United States; ^15^ IDC Instituto de Cáncer Hemato Oncólogos, Cali, Colombia; ^16^ Prodigy Cells Labs, LLC, Doral, Florida, United States

**Keywords:** CAR-T therapy, nanotechnology, artificial intelligence, machine learning, deep learning, immunotherapy, manufacturing, personalized medicine

## Abstract

Personalized medicine has redefined cancer treatment by aligning therapies with each patient’s unique biological profile. A key example is chimeric antigen receptor T-cell (CAR-T) therapy, in which a patient’s own T cells are genetically modified to recognize and destroy cancer cells. This approach has delivered remarkable results in hematologic malignancies and is beginning to show promise in solid tumors and autoimmune diseases. However, its broader adoption is limited by major challenges, including complex manufacturing, high costs, limited efficacy in solid tumors, and potentially severe toxicities. Nanotechnology offers exciting possibilities to overcome many of these barriers. Engineered nanoparticles can improve gene delivery, target tumors more precisely, enhance immune cell function, and enable *in vivo* CAR-T production, reducing the need for labor-intensive *ex vivo* processes. However, despite this promise, translation into clinical settings remains difficult due to regulatory hurdles, scalability issues, and inconsistent reproducibility in human models. At the same time, artificial intelligence (AI), with its powerful algorithms for data analysis and predictive modeling, is transforming how we design, evaluate, and monitor advanced therapies, including the optimization of manufacturing processes. In the context of CAR-T, AI holds strong potential for better patient stratification, improved prediction of treatment response and toxicity, and faster, more precise design of CAR constructs and delivery systems. Leveraging these three technological pillars, this review introduces the concept of *Smart CART Nanosymbionts*, an integrated framework in which AI guides the design and deployment of nanotechnology-enhanced CAR-T therapies. We explore how this convergence enables optimization of lipid nanoparticle formulations for mRNA transfection, specific targeting and modification of the tumor microenvironment, real-time monitoring of CAR-T cell behavior and toxicity, and improved *in vivo* CAR-T generation and overcoming barriers in solid tumors. Finally, it’s important we also address the ethical and regulatory considerations surrounding this emerging interface of living therapies and computational driven systems. The *Smart CART Nanosymbionts* framework (**Figure 1**:) represents a transformative step forward, promising to advance personalized cancer treatment toward greater precision, accessibility, and overall effectiveness.

## 1 Introduction

Personalized medicine is redefining approaches to cancer prevention, diagnosis, and treatment by leveraging each patient’s unique biological profile (1). Among its most significant advancements is CAR-T cell therapy, which involves genetically reprogramming a patient’s own T cells to recognize and eliminate cancer cells expressing specific antigens (2). Since the first approval by the U.S. Food and Drug Administration (FDA) in 2017, CAR-T cell therapy has provided critical treatment options for patients with recurrent leukemias, lymphomas, and myelomas, achieving unprecedented remission rates when conventional treatments have failed (3–6). In the field of autoimmunity, its benefits are beginning to be demonstrated mainly in systemic lupus erythematosus (7).

Despite its successes, several serious challenges impede broader adoption. Reliance on viral vectors for gene delivery introduces risks, including variable transfection efficiency, stringent regulatory requirements, and potential insertional mutagenesis (8–10). Such concerns have prompted regulatory agencies like the FDA to emphasize the urgent need for safer and more precise gene delivery methods (11). Off-target cytotoxicity also remains a major issue, manifesting in severe complications such as cytokine release syndrome (CRS), macrophage activation syndrome (MAS)/hemophagocytic lymphohistiocytosis (HLH), and neurological toxicities. Furthermore, CAR-T therapy is less effective in solid tumors due to immunosuppressive microenvironments and physical barriers that limit T-cell infiltration and function (8, 12, 13). It also comes with high expenses, almost $400,000 per patient and approaching $1 million with associated care (14–16).

Nanotechnology is emerging as a promising solution to these challenges. It enhances CAR-T cell persistence, infiltration, functionality and can provide non-viral gene delivery platforms. It also facilitates real-time monitoring of CAR-T cell activity and supports *in vivo* CAR-T generation, reducing the need for labor-intensive *ex vivo* processes (17–21).

**Figure 1:**
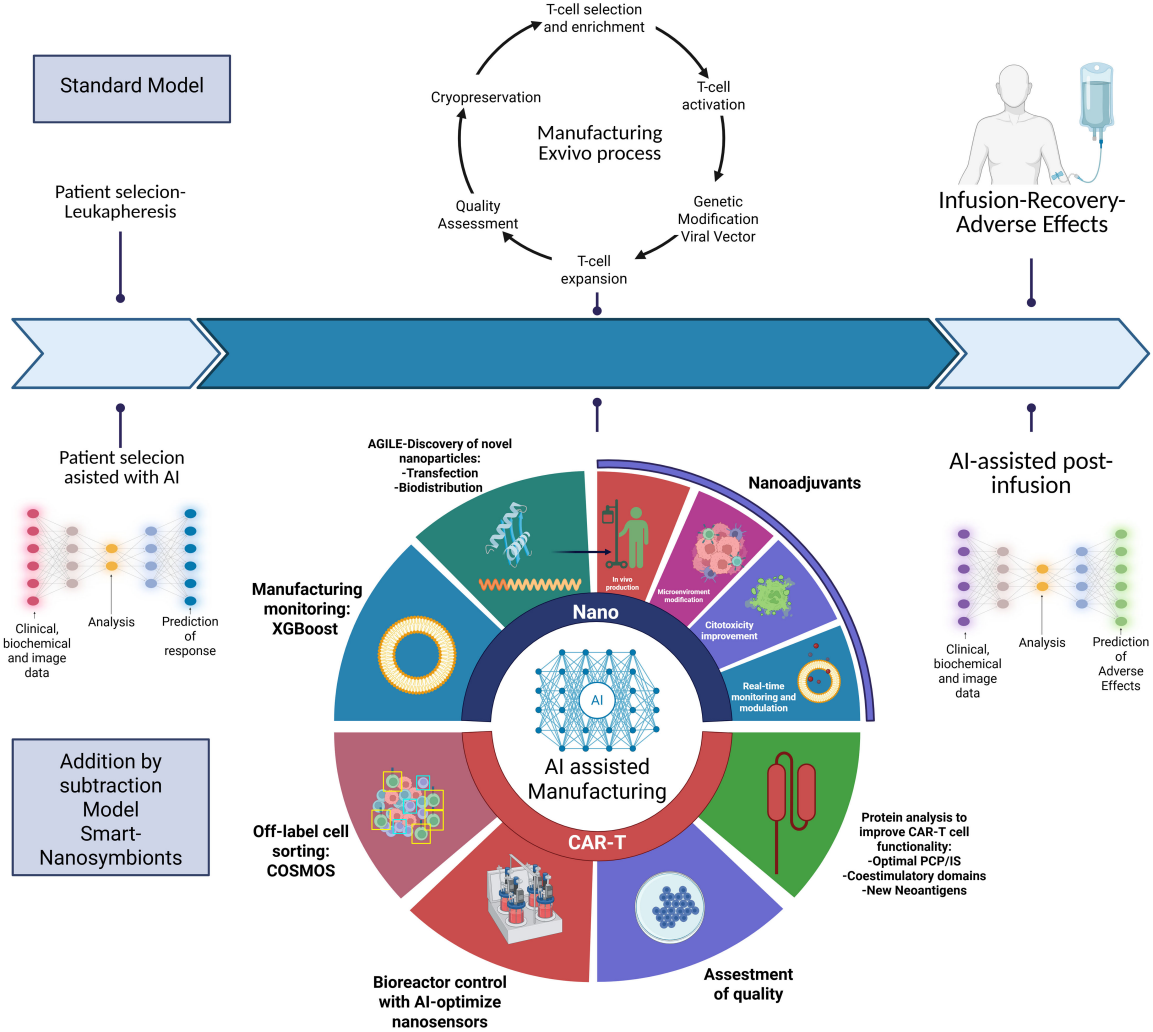
Compares the use of ML and DL in optimizing the production of advanced therapies, such as CAR-T cells and nanoparticles. In the ML section the algorithm can be trained for feature extraction, incorporating clinical data (such as biomarkers and immune responses) and manufacturing data (such as ethanol concentration and total flow rate). These inputs are analyzed to enable early prediction of CRS and ICANS toxicity and to improve the efficiency and quality of nanoparticle production. The DL section presents a more advanced approach using, ANNs, CNNs and GNNs. These technologies analyze proteins, RNA sequences, cellular imaging, and spatial representations. Key applications include the design of nanoparticles with enhanced tumor penetration, the optimization of CAR-T cells with greater cytotoxicity and persistence(CAR-Toner and motifs analysis), and the classification of cells based on their sensitivity or resistance to CAR-T treatment. Additionally, image-based predictions help assess cell sorting (COSMOS), therapeutic response and immune synapse quality. CRS, Cytokine Release Syndrome; ICANS, Immune Effector Cell-Associated Neurotoxicity Syndrome; ANNs, Artificial Neural Networks; GNNs, Graph Neural Networks.

Even with this new perspective, there are still cogs in the machine that could be better integrated. AI can streamline manufacturing workflows, automate complex data analyses, and refine the design for nano-driven CAR-T systems, enhancing both efficiency and effectiveness. Defined as the development of systems capable of performing tasks traditionally requiring human intelligence, AI employs advanced algorithms and self-learning models that adapt, identify patterns and make autonomous decisions (22–24). The integration of AI would allow for smart tuning of nanoparticle properties, such as size and surface charge, to optimize delivery efficiency and safety and in the context of CAR-T. Additionally, AI supports CAR-T optimization through predictive tools for patient selection, receptor design, cell classification and quality control, and early toxicity prediction. In this review, we explore how the fusion of nanotechnology and artificial intelligence can help address the current challenges facing CAR-T therapy and describe current AI-models applied to specific problematics. This approach has the potential to revolutionize how CAR-T therapy and nanotechnology come together, signaling a major leap forward in precision in oncology. We introduce a model we call Smart CAR-T Nanosymbionts and combined with the previous Addition by Subtraction model we authors proposed in previous reviews (**Figure 1**). A model designed to streamline the treatment of patients by reducing inefficiencies, lowering costs, and enhancing therapeutic outcomes through AI-powered nanotech in the context of pathologies targetable with CAR-T. This conceptual framework guides the structure of the review and offers a future-oriented lens for understanding how these technologies can reshape CAR-T innovation (25–28). We have created a glossary (**Table 1**) at the end of this paper for guidance.

**Table 1:** Glossary.

Term	Definition
Advanced Therapies and Nanotechnology	
Nanosymbiont	A nanoparticle or nanoscale object that interacts symbiotically with cells or biological systems.
LNP	Lipid Nanoparticle. Lipid-based nanoparticles used to deliver mRNA.
PEG	Polyethylene Glycol. A polymer that enhances nanoparticle stability and avoids immune recognition.
SPION	Superparamagnetic Iron Oxide Nanoparticles. Used for magnetic guidance and imaging-based monitoring.
Nanozyme	A nanoparticle with catalytic properties similar to natural enzymes.
Nanobodies (VHH)	Antibody fragments derived from camelids, smaller and more stable than conventional antibodies.
scFv	Single-chain Variable Fragment. An antibody fragment commonly used in CAR receptors.
PdI	Polydispersity Index. A measure of size distribution in nanoparticles.
Artificial Intelligence and informatics	
AI	Artificial Intelligence. Algorithms that emulate human intelligence.
ML	Machine Learning. A branch of AI that learns from structured data.
DL	Deep Learning. An advanced subfield of ML using deep neural networks.
CNN	Convolutional Neural Network. A DL model used for image analysis.
RNN/LSTM	Recurrent Neural Network/Long Short-Term Memory. Neural networks for analyzing sequential data.
GNN	Graph Neural Network. Analyzes structured data such as protein-protein interactions.
XGBoost	A decision tree-based algorithm used for high-accuracy prediction and classification.
AGILE	AI-Guided Ionizable Lipid Engineering. A DL platform for discovering optimal lipids for mRNA delivery.
MHCnuggets	A DL algorithm that predicts peptide binding to MHC molecules, useful for neoantigen discovery.
NbAffinity	An ML platform for predicting affinity between nanobodies and their target ligands.
CAR-TONER	An AI tool that optimizes the structural design of CAR receptors, particularly positive charge patches (PCPs).
COSMOS	Computational Sorting and Mapping of Single Cells. A DL system for label-free cell classification.
RCMNet	A neural network for precise identification of CAR-T cells in peripheral blood.
M2-CRS	An SVM-based ML model for predicting the risk of cytokine release syndrome (CRS).
SHAP	SHapley Additive exPlanations: method used to interpret machine learning models by quantifying the contribution of each input feature
SERS	Surface-Enhanced Raman Scattering: highly sensitive spectroscopic technique that amplifies Raman signals of molecules when they are in close proximity to nanostructured metal surfaces (such as gold or silver nanoparticles).
MOBO	Multi-Objective Bayesian Optimization. An algorithm for optimizing multiple variables simultaneously.
Cell Biology and Immunotherapy	
CRS	Cytokine Release Syndrome. A common immune-related complication in CAR-T therapy.
ICANS	Immune Effector Cell-Associated Neurotoxicity Syndrome. Neurotoxicity related to CAR-T therapy.
TME	Tumor Microenvironment. The microenvironment around the tumor, which may hinder immune efficacy.
TAMs	Tumor-Associated Macrophages. Macrophages within the TME that suppress immune responses.
MDSCs	Myeloid-Derived Suppressor Cells. Myeloid cells that inhibit immune response.
IL-15/IL-12	Interleukins used to boost T cell expansion and persistence.
TGF-β	Transforming Growth Factor Beta. An immunosuppressive molecule in the TME that inhibits CAR-T cell activity.
Manufacturing and Optimization	
GMP	Good Manufacturing Practice. Quality standards for drug and therapy production.
Bioreactor	A system used to culture and expand T cells under controlled conditions.
Pd/Pt-glucose sensor	A glucose sensor based on palladium/platinum for metabolic control in cell cultures.
ANS	Automated Nanoparticle Synthesizer. A robotic platform for autonomous nanoparticle production.

## 2 CAR T-Cell Nanosymbionts: redifying the frontier of cellular immunotherapy with nanotech synergy

A nanosymbiont refers to a nanoscale entity that forms a symbiotic relationship with a host system, typically at the cellular or macromolecular level. These nanosymbionts are often engineered nanoparticles or smarter nano-devices—such as nanomachines and nanorobots—that interact beneficially with biological systems by mimicking or enhancing natural symbiotic functions. These nanosymbionts are often made from lipid-based nanostructures (like liposomes), polymeric nanoparticles, DNA/RNA nanostructures (e.g., nanosnowflakes) (259), Inorganic nanomaterials (e.g., gold or silica nanoparticles) (260).

The discovery of materials exhibiting unique and distinctive properties at the nanoscale (generally defined as structures with dimensions smaller than 100 nanometers) has led to the development of the multidisciplinary field of nanotechnology. This field encompasses the development, synthesis, characterization, and utilization of nanomaterials and nanodevices across various disciplines, including environmental science, electronics, energy, and medicine (261).

Nanoparticles possess a diverse array of tunable properties. These include customizable chemical compositions and internal structures, high surface-to-volume ratios, defined geometries, and distinctive electrical, magnetic, optical, and catalytic behaviors (e.g., nanozymes) (29–32). Their surfaces can be further engineered with functional ligands—such as pH-responsive polymers, polyethylene glycol (PEG), lipids, and biomolecules including antibodies and aptamers, to improve encapsulation efficiency, enhance bioavailability, and enable targeted interactions with biological systems (33–35). These characteristics make nanoparticles especially valuable in biomedical applications, notably for drug delivery. Their small size and surface engineering capabilities enable enhanced permeability and retention (EPR) effects, as well as controlled and sustained drug release. These features have significantly advanced their use in cancer therapy and diagnostics (18, 36–39). Moreover, nanoparticles can be designed to circumvent drug resistance—an obstacle commonly encountered in conventional chemotherapy. By conjugating ligands that selectively bind to receptors overexpressed on cancer cells, nanoparticles can deliver therapeutic agents directly to tumor sites (smart drug delivery systems). This targeted delivery minimizes collateral damage to healthy tissues, improves therapeutic efficacy, and enhances imaging precision (36, 40–44). Consequently, nanotechnology provides innovative strategies to enhance the efficacy, specificity, and safety of cancer treatments, offering potential cost-effective alternatives to traditional methodologies (45, 46). Synthetic nanomachines (nanoconverters, Janus nanomotor, etc.) or nanorobots (ultrasound-propelled biomimetic nanorobot) introduced into cells to perform functions like detoxification, sensing, or energy generation (47–49). Also, nanoparticles that help reprogram immune cells (like CAR-T cells) or interact with microbiota to influence host health.

This field is transforming CAR T-cell design by creating precise, multifunctional platforms that address key therapeutic challenges. For instance, lipid-based and polymer nanoparticles have been developed to deliver mRNA encoding CAR constructs directly into T cells, circumventing safety concerns associated with viral vectors and ensuring efficient delivery with reduced immunogenicity (50, 255). Furthermore, nanotechnology plays a crucial role in developing *in vivo* CAR T-cell therapies, wherein nanoparticles deliver CAR-encoding materials directly to circulating T cells within patients (51–54). Additionally, nanomachines can selectively target different lymphocyte subtypes via surface-attached antibodies, facilitating precise genetic material delivery. Virus-like nanoparticles engineered for systemic gene therapy demonstrate prolonged circulation time, reduced immunogenicity, and efficient and safe gene delivery to target cells (18).

There are numerous stages in the development of CAR-T cells and aspects of tumor biology where nanotechnology can be effectively integrated. The following are examples of how these two technologies have been successfully combined. The immune synapse (IS) is crucial for car t cell activation, triggering cytotoxic T lymphocytes (CTLs) and CAR-IS shows higher expression of molecules with antiapoptotic and antiproliferative (55, 56). To potentiate this effect, agents like histone deacetylase inhibitors (HDACi), such as panobinostat, can enhance Fas-mediated apoptosis, improving antitumor efficacy (57). Nanocarriers, such as poloxamer 407-based nano-micelles, enhance intracranial delivery of panobinostat in glioma models, increasing therapeutic concentration and tumor response (56).

Precise interaction between the CARs and tumor cell epitopes is essential for CAR T cell function. The scFv fragment, the CAR’s antigen-binding domain, can suffer from misfolding, aggregation, and overstimulation of T cells, leading to early exhaustion. Nanobodies, small, single-domain antibodies derived from camelids, due to their compact size, high stability, and reduced immunogenicity can avoid misfolding and aggregation ensuring a more controlled activation of CAR T cells, preventing the overexpression of cytotoxic signals that could prematurely exhaust the T cells. Nanobodies improve antigen recognition, leading to a stronger and more durable synapse between the CAR T cells and tumor cells. They can also serve as modular structures that facilitate the redirection of universal CAR T cells to target various tumor antigens, further enhancing the precision and adaptability of the therapy (55).

In solid tumors, interferon-γ receptor (IFNγR) signaling is critical for cell adhesion after CAR T cell treatment and its impairment can derive in CAR T cell binding reduction and resistance (58).

SCH-58261-loaded cross-linked multilamellar liposomes with maleimide functionalization on the surface of CAR T cells have been designed in models of ovarian cancer and chronic myelogenous leukemia to target the A2a adenosine receptor (A2aR) inhibitory pathway involved in T cell receptor signaling inhibition and IFNγ production through elevated intracellular cyclic AMP adenosine levels that are increased in the tumor microenvironment. These liposomes enhance the colocalization of nanoparticles in sensitive tumor areas and prolonging tumor growth inhibition by targeting the A2a receptor pathway (59).

To broaden the targeting landscape, aptamers (short, single-stranded nucleic acids capable of binding to target cancer cells with high specificity and affinity) are functionalized with nanocarriers to deliver cytokines, immune checkpoint inhibitors, or cytotoxic agents directly to the tumor site. This expands the antigenic repertoire beyond conventional antibody recognition. The SELEX (Systematic Evolution of Ligands by Exponential Enrichment) technique enables identification of high-affinity aptamers, which, when combined with nanodevices, enhance immune synapse formation and therapeutic delivery (60).

T cell exhaustion, one of the main concerns regarding efficacy and resistance in CAR T cell therapy, occurs due to increased inhibitory signals from molecules such as PD-1, Tim-3, LAG-3, VISTA, CTLA-4, and TIGIT, affecting both tumor cells and T lymphocytes (61). Magnetic nanoclusters, equipped with PD-1 antibodies, utilize a pH-sensitive bond for attachment and bind to effector T cells through PD-1 receptors. In an acidic environment, they release anti PD-L1 antibodies, blocking PD-1 interactions and maintaining CTL functionality above 90% while delaying tumor progression. The treatment also reduced the abundance of Tregs and increased the abundance of CD8+ CTLs in tumor-bearing mice (62). Moreover, the conjugation of liposomes and synthetic nanoparticles with CD8+ T lymphocytes via maleimide-thiol coupling provides continuous pseudoautocrine stimulation of transferred cells. Following this rational, some research groups have developed multilamellar lipid nanoparticle core loaded with IL-15 and IL-21 to release cytokines in very low doses over several days resulting in significantly higher proliferation compared to systemic infusion (63).

Epitope spreading arises because of residual dying tumor cells are captured and processed by APCs. This leads to the presentation of novel peptides via MHC class I and II molecules, thereby priming naïve T cells to recognize and attack tumor-associated antigens distinct from those originally targeted by CAR T cells. This secondary immune activation broadens the anti-tumor response beyond the specificity of the initial CAR construct. Tumor cryptic antigens must be presented on MHC Class I molecules, engaging cytotoxic CD8+ T cell responses via cross-presentation, primarily performed by specific APCs (64). Dendritic mesoporous silica nanoparticles (DMSNs), modified with hyaluronic acid and covalently bound to anti-CD3, anti-CD28, and anti-PD-1, facilitate T cell activation and antigen cross-presentation, enhancing IFN-β secretion and MHC upregulation and reducing the likelihood of the cells escaping immune surveillance (65). Additionally, dendritic cell-biomimetic nanoparticles and dendritic cell-mimicking nanovaccines (nanoDCs), fabricated from mature bone marrow-derived DC membranes loaded with TAAs improve CD8+ T cell priming, reduce tumor size, and inhibit metastasis (66, 67). In parallel, other approaches have explored the use of lipopolyplex platforms, in which mRNA molecules are encapsulated within a polymeric polyplex core and subsequently enclosed in a phospholipid bilayer shell. This architecture enables the delivery of mRNA encoding multiple tumor-associated antigens, while concurrently acting as an adjuvant by engaging Toll-like receptor (TLR) signaling in APCs. These nanoconstructs have demonstrated efficiente uptake by dendritic cells, promoting robust antigen presentation, and the bilayer shell effectively prevents nonspecific interactions of the mRNA core with non-target cells, thereby reducing off –target effects and systemic toxicity (68).

Moreover, the tumor microenvironment—through stromal and myeloid cells, angiogenesis, and cytokines like TGF-β—impairs CAR T cell trafficking, infiltration, and cytotoxic function, significantly limiting their efficacy in solid tumors (69). Nano-backpacks utilize T lymphocytes as vehicles to take loaded nanoparticles to tumor microenvironment. Liposomes loading a potent small molecule that works as an inhibitor of the TGF-β receptor I restore granzyme B expression to a higher level than systemic TGF-β inhibitors. Also, these liposomes promoted division and expansion of T cells (70). In line with other backpacking strategies, chemically tethering an interleukin-15 superagonist to T cells via drug-releasing protein nanogels anchored to CD45 has shown enhanced intratumoral expansion. These nanogels are activated upon antigen engagement within the tumor microenvironment, resulting in a 16-fold increase in T cell proliferation compared to systemic cytokine delivery, and over 1,000-fold compared to T cells lacking cytokine support (71).

Other components of TME such as fibrotic stroma and compressed vasculature can be bypassed through photothermal strategies. CAR T cells engineered with indocyanine green nanoparticles (CT-INPs) generate localized heating upon NIR exposure, causing 98% tumor cell death and promoting vasodilation and immune infiltration (72). In addition, vascular-targeted nanorobotics offers a radical approach. DNA origami nanobots loaded with thrombin selectively induce tumor thrombosis by releasing their cargo upon receptor binding, starving the tumor (73).

Strategies currently used by clinicians to follow activity of infused CAR T cells are invasive and do not provide real-time whole-body spatio-temporal distribution of infused T cells. There is a clinical need for a technique that can monitor *in vivo* performance of CAR T cells in tumors and off-target sites. Ferumoxytol (iron oxide nanoparticles detected by MRI) can be used as a cell marker to monitor real time *in vivo* CAR T cell in preclinical osteosarcoma model. These CAR T cells are identified through MRI, photoacoustic tomography (PAT), and magnetic particle imaging (MPI). Tumor demonstrates iron enhancement on T2-weighted MRI only in the ferumoxytol-labeled B7-H3 CAR T cell group indicating enhanced infiltration of the T cells in the tumor tissue (20). Similarly, CAR T cells co-expressing CD19 and luciferase can be radiolabeled with gold nanoparticles functionalized with copper-64 and PEG (GNP-64Cu/PEG2000), allowing long-term PET imaging that correlates with bioluminescent signal, confirming the possibility of tracking CAR T cells using positron emitter imaged by PET/CT scanner (21).

Finally, the conventional design of CAR T cells may elicit unpredictable toxicities due to their inherent capacity for expansion, persistence, and recognition of both malignant and non-malignant cells expressing tumor-associated antigens. Reversible control of CAR T activity has been demonstrated using the tyrosine kinase inhibitor dasatinib (DAS), which blocks CD3ζ ITAM phosphorylation, thereby suppressing CAR T effector function and mitigating acute toxicities. However, DAS presents pharmacokinetic limitations, including poor water solubility, pH-dependent absorption, and a short half-life (3–4 h). To address this, a pH-sensitive nanoparticle linking DAS to hyaluronic acid (DAS-HA) enables targeted release in the acidic tumor microenvironment, enhancing intracellular DAS accumulation and improving the safety and efficacy of CAR T modulation (74, 75).

**Figure 2:**
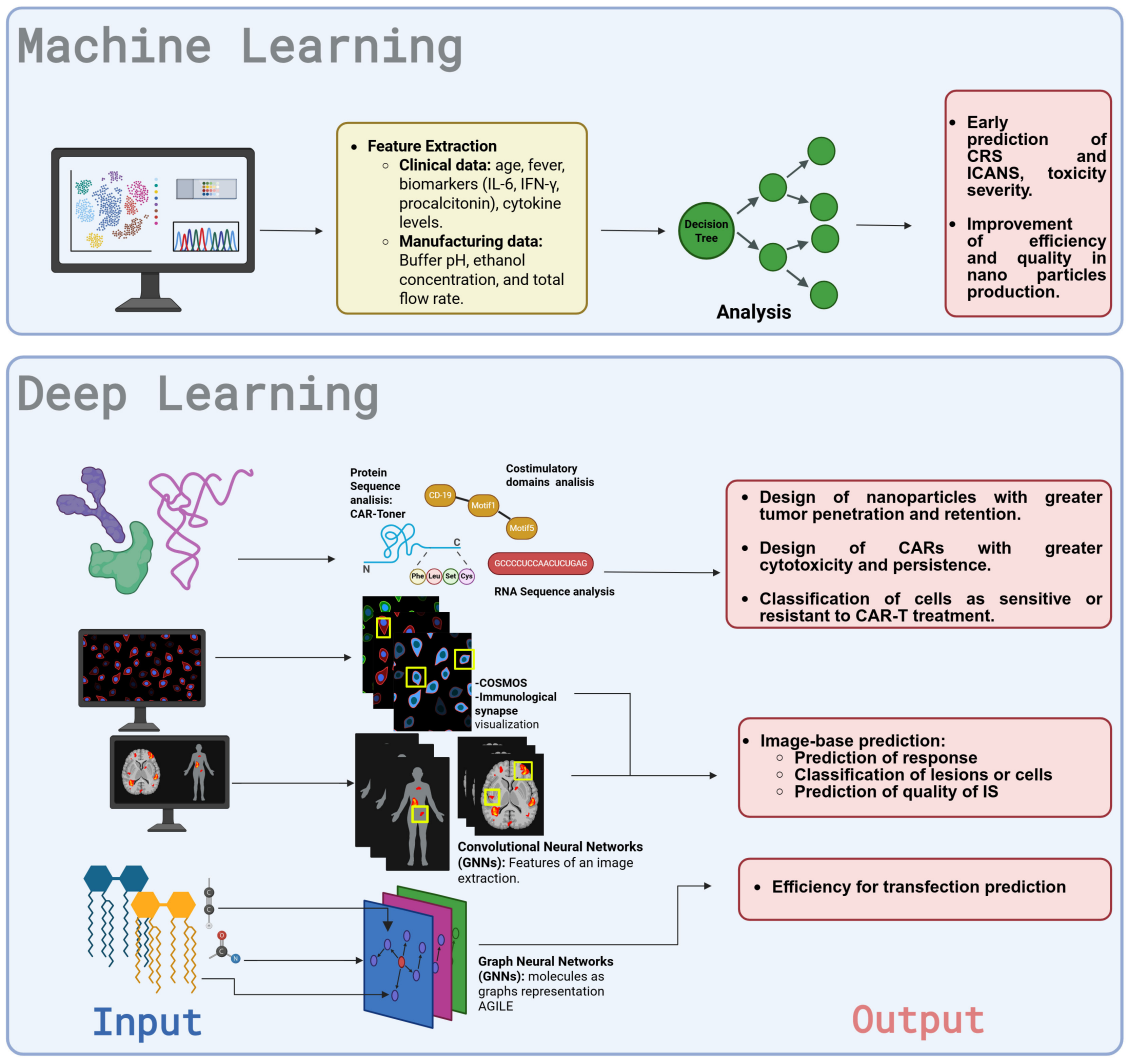
The conventional CAR-T cell manufacturing model involves a complex *ex vivo* process with multiple steps, including leukapheresis, T-cell selection, genetic modification using viral vectors, expansion, and cryopreservation before infusion, leading to high costs, long production times, and logistical challenges. The “Addition by Subtraction Model: Smart CAR-T Nanosymbionts “ model integrates AI and nanotechnology to streamline production by reducing process steps, replacing viral vectors with non-viral alternatives (e.g., nanoparticles), and leveraging *in vivo* genetic modification to enhance efficiency. AI-driven patient selection analyzes clinical, biochemical, and imaging data to predict response, while AGILE-based discovery of nanoparticles optimizes transfection and biodistribution for improved CAR-T functionality. AI-enhanced bioreactor control using nanosensors ensures real-time monitoring and quality assessment and technologies like COSMOS helps in label-free sorting cells, refining the final CAR-T product. Additionally, AI-driven protein analysis optimizes CAR structure by improving peptide-CAR interactions, refining co-stimulatory domains, and identifying new neoantigens to enhance efficacy. Post-infusion, AI assists in predicting and managing adverse effects, while nanoadjuvants dynamically regulate CAR-T function, mitigating toxicity, preventing exhaustion, promoting epitope spreading, and strengthening the immunological synapse (IS). This AI- and nanotechnology-driven approach enhances CAR-T therapy by improving safety, reducing costs, and increasing accessibility, marking a significant advancement in cancer immunotherapy. Created with BioRender.com.

Summary of the applications described in the text are found in **Table 2** and Graphical representation in **Figure 2**:

**Table 2:** Nanotechnology application in CAR-T therapy.

Category	Nano Strategy	Description
Enhanced CAR-T Response and Persistence	Nanobody replacement of scFv	Smaller and more stable fragments reduce immunogenicity and improve CAR-T specificity (76)
	HDACi in nanocarriers	Histone deacetylase inhibitors reinforce immune synapse and CAR-T activity (77)
	Bioengineered polymer matrices	Controlled release of co-stimulatory signals to maintain CAR-T function (78)
	Anti-PD-L1 nanocarriers	Blocking PD-1 interactions to prevent CAR-T dysfunction (61)
	Pseudoautocrine stimulation	Nanoparticles provide continuous activation signals to CAR-T cells (79)
Improved Tumor Infiltration and Adhesion	Multilamellar liposomes	Facilitate CAR-T localization in tumors, enhancing adhesion and activation (72)
	Aptamer-functionalized nanocarriers Incorporating bioactive phospholipids into LNP formulations	Direct CAR-T to multiple tumor antigens, improving interaction with cancer cells (60) Different phospholipids can alter the interaction of LNPs with immune cells, potentially improving adhesion to tumor sites (80)
Overcoming Tumor Escape	mRNA lipopolyplexes	Deliver mRNA encoding multiple tumor antigens to prevent immune evasion (68)
	Dendritic mesoporous silica nanoparticles	Promote antigen spreading and enhance immune response (66, 67, 81)
Tumor Microenvironment Modulation	TGF-β inhibitor liposomes	Block TGF-β signaling to restore CAR-T cytotoxicity (70, 82)
	Protein nanogels with IL-15	Localized CAR-T expansion in tumors without systemic toxicity (83)
	NIR-activated nanoparticles	Generate localized heat to improve immune infiltration and destroy tumor cells (84)
Gene Delivery and Manufacturing Optimization	Liposomes carrying mRNA	Temporarily induce CAR receptor expression in tumor-infiltrating lymphocytes (85)
	Gene-carrier nanoparticles	Reprogram T cells *in vivo*, eliminating the need for *ex vivo* manufacturing (54, 86)
	Microfluidic and nanobioreactors	Optimize CAR-T expansion to reduce costs and improve quality (87)
Monitoring and Toxicity Reduction	Iron oxide nanoparticles	Real-time CAR-T tracking with imaging (20, 88)
	Gold nanoparticles with Cu-64	
	pH-sensitive nanoparticles	Drug release only in acidic tumor environments to reduce systemic toxicity (74)
	Nanoparticles with Dasatinib	Regulate CAR-T activation outside tumors to minimize adverse effects (75)

scFv, Single-chain Variable Fragment; HDACi, Histone Deacetylase Inhibitors; PD-1/PD-L1, Programmed Death-1/Programmed Death Ligand-1; LNP, Lipid Nanoparticle; TGF-β, Transforming Growth Factor Beta; IL-15, Interleukin 15; NIR, Near-Infrared Radiation; MRI, Magnetic Resonance Imaging; PET/CT, Positron Emission Tomography/Computed Tomography.

## 3 Machine intelligence in motion: from algorithms to autonomy

AI encompasses a wide range of subfields and methodologies aimed at replicating or augmenting human-like intelligence in machines (89). Rather than representing a single technology, AI consists of a diverse set of interconnected tools and systems with vast potential, particularly in the healthcare domain (90). One of its most prominent branches is machine learning (ML), which focuses on developing systems capable of learning from historical data. These systems leverage algorithms trained on context-specific features within structured datasets to identify patterns and generate predictions, using approaches such as supervised, unsupervised, or reinforcement learning (24, 91). In healthcare, ML is commonly applied to structured medical data for tasks such as early disease detection, prediction of therapeutic responses, and optimization of clinical resource allocation (92, 93). Within ML, deep learning (DL) represents an advanced subcategory that employs artificial neural network-based algorithms to process and analyze large-scale datasets with complex structures (94). Unlike traditional ML techniques, DL excels at uncovering intricate correlations that are often undetectable using conventional methods. One of its core advantages lies in its ability to autonomously perform feature extraction from unstructured data such as medical imaging, genetic sequences, or clinical text, thereby eliminating the need for manual preprocessing. These capabilities have made DL particularly valuable in areas such as drug discovery and the detection of cancerous lesions in radiological images (92, 95, 96).

**Figure 3:**
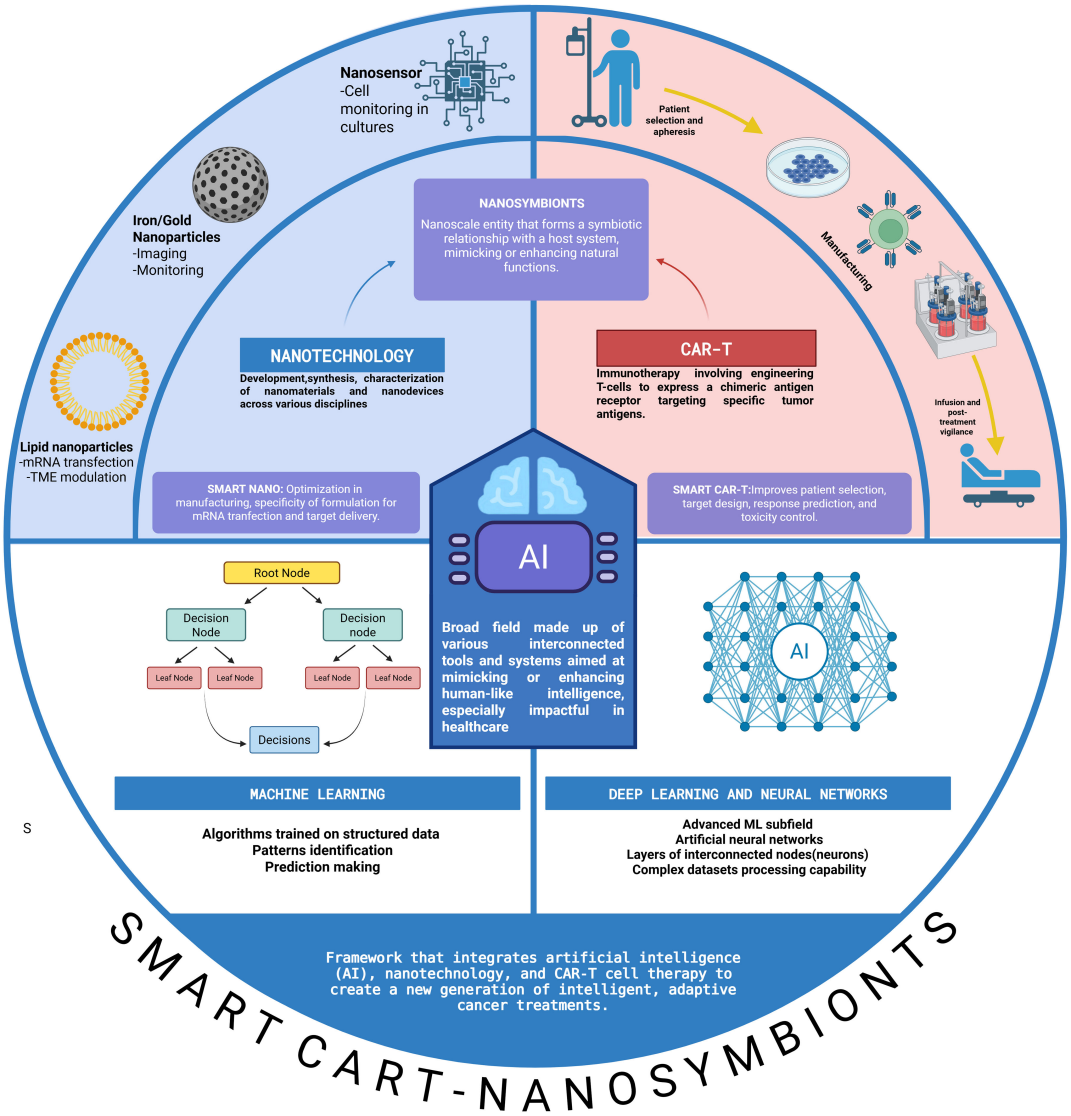
Schematic representation of lipid, polymer, inorganic, and hybrid hydrophobic polymeric nanoparticles (NPs) and the possible advantages of using nanotechnology in CAR T cells (1): Tumor microenvironment remodeling: using indocyanine green nanoparticles plus infrared light irradiation to disrupt the ECM before CAR administration, using targeted nanocarriers with *in vitro* transcribed mRNA to reprogram TAMs and downregulate PD-L1, and using nanozymes and nanoparticle backpacks. (2) Improving T cell proliferation and lifespan with mesoporous silica micro-rods secreting IL-2, APC cell-membrane mimics, using RNA-LPX to activate T cells, and NPs linking APCs to prime and activate T cells. (3) Improving follow-up and resistance with genetic programming using mRNA nanocarriers for targeted gene expression and NBiTE generation and radiolabeled NPS to track T cells *in vivo*. APC, antigen-presenting cell; ECM, extracellular matrix; IL-2, interleukin-2; NBiTEs, nano-bispecific T cell engagers; NPs, nanoparticles; PD-L1, programmed cell death ligand-1; TAMs, tumor-associated macrophages. Created with BioRender.com. Taken with permission from: Baena JC, Pérez LM, Toro-Pedroza A, Kitawaki T, Loukanov A. CAR T Cell Nanosymbionts: Revealing the Boundless Potential of a New Dyad. Int J Mol Sci. 2024 Dec 7;25(23):13157. doi: 10.3390/ijms252313157. PMID: 39684867; PMCID: PMC11642191.

Among the algorithms encompassed within DL are convolutional neural networks (CNNs) and recurrent neural networks (RNNs), which are designed to replicate biological processes for data analysis. CNNs process visual data by extracting local features at various levels of depth, enabling the identification of complex patterns in images (97, 98). These representations are typically complemented by Multilayer Perceptron (MLP) layers (fully connected neural networks), which are responsible for performing the final classification based on the extracted features (99). In contrast, RNNs—including Long Short-Term Memory (LSTM) networks—capture temporal dependencies in tasks such as language modeling, DNA sequence analysis, and continuous time series prediction, making them valuable for both classification and regression problems (100). These architectures enhance drug–target interaction prediction, pharmacokinetic modeling, and toxicity assessment (101, 102), addressing the high expenses, time demands, and ethical concerns of traditional experimental methods (101, 103). A summary of the current AI-models utilized in healthcare are found in **Table 3**. Integrating AI with CAR-T therapy and nanotechnology presents remarkable opportunities for enhancing treatment design, delivery, and development, driving advancements in personalized medicine. Current advances in AI for nanotechnology and CAR-T therapy, independently, are shown in **Figure 3**.

**Table 3:** Artificial Intelligence types and their applications.

Type of AI	Key Characteristics	Example Use Cases
Machine Learning (ML) (24, 91, 93)	Algorithms that identify patterns in historical data to generate predictions. They work mainly with structured data containing relevant contextual features. By learning from past examples, they generalize to new, unseen cases.	• Pathology classification • Epidemiological pattern detection • Diagnosing failures in complex systems • Recommendation systems
Deep Learning (DL) (92, 95)	Advanced ML subset using neural networks inspired by the brain. Extracts complex features from large, often unstructured data. Requires high computational power(GPUs or TPUs)	• Bioinformatics • Drug design • Computer vision • Medical imaging • Natural language processing
Convolutional Neural Networks (CNNs) (98)	Deep learning model inspired by the visual cortex, optimized for visual and spatial data.Uses convolutional layers to capture local patterns and spatial hierarchies.	• Image classification • Object detection/segmentation • Facial recognition • Medical imaging analysis • Pattern recognition
Recurrent Neural Networks (RNNs) (100)	Deep-learning architecture specifically engineered for sequential or time-series data, with neurons maintaining a ‘memory’ through feedback loops.	• Language modeling • Text generation (chatbots, predictive typing) • Physiological time-series forecasting • Genomic sequence analysis • Biomedical signals interpretation • Pharmacokinetic modeling
Graph Neural Networks (GNNs) (104)	Models relational and graph-structured data (nodes and edges), capturing interactions between biological entities (e.g., proteins, drugs).	• Protein–protein interaction networks, biological pathways • Multi-omic data integration • Drug repositioning
Generative Models (GANs, Diffusion Models) (105)	Models trained to learn data distributions and generate synthetic samples, including GANs, VAEs, DDPMs, and LLMs like GPT.	• Synthetic patient data generation • Augmenting rare disease datasets • Virtual clinical trial simulations
Transformers (106)	Attention-based architecture excelling in sequential and contextual data analysis. It can vercomes long-range dependency limitations of RNNs and applicable to both discriminative (classification) and generative (text/image generation) tasks.	• Clinical natural language processing (e.g., radiology report summarization) • Patient outcome prediction • Drug–target interaction modelling

**Table 3**. Overview of artificial intelligence techniques: Highlighting key characteristics and representative applications, emphasizing their unique architectures, computational requirements, and suitability for specific tasks across various domains. GANs, Generative Adversarial Networks; VAEs, Variational Autoencoders; DDPMs, Denoising Diffusion Probabilistic Models; LLMs, Large Language Models; GPT, Generative Pre-trained Transformers.

## 4 Synergy of code and cell: AI-powered revolution of CAR-T cell therapies

The integration of AI into CAR-T cell therapies has opened a transformative avenue toward precision oncology, significantly enhancing therapeutic outcomes through optimized patient selection, streamlined manufacturing, CAR design optimization, and accurate prediction of adverse effects. By manipulating advanced computational models and deep learning algorithms, AI addresses critical limitations within current CAR-T cell protocols, such as inconsistent patient responses, complex manufacturing processes, receptor optimization challenges, and treatment-associated toxicities. This section systematically reviews current evidence and applications demonstrating how AI-driven solutions for CAR-T therapy

### 4.1 Patient selection

Accurate patient selection is essential for the successful implementation of advanced therapies such as CAR-T cell treatment. AI improves patient selection by integrating and analyzing clinical data, such as demographic variables, laboratory results, imaging biomarkers, to identify complex, non-linear correlations that often go unnoticed by conventional statistical methods. By training on large volumes of data, AI models can learn patterns that link specific patient characteristics with therapeutic response or toxicity risk. This enables the stratification of patients based on response or clinical risk, supporting more personalized and evidence-based decision-making (107, 108).

In the pivotal JULIET trial, patients with relapsed or refractory diffuse large B-cell lymphoma (DLBCL) who received a single infusion of Tisagenlecleucel demonstrated a 53% overall response rate (ORR) and a 39% complete response rate (CR). However, significant challenges persisted, evidenced by a median progression-free survival (PFS) of only 2.9 months, with 62% of patients ultimately experiencing disease progression or death. To address these limitations, secondary analyses utilized AI to enhance predictive outcomes and patient stratification. Integrating pre-infusion FDG PET/CT imaging with clinicopathological data, AI models, particularly attention-gated AG-CNNs, were applied to delineate disease foci and improve outcome predictions. The AI analysis revealed that 52.4% of patients exhibited a negative AI signature, and all patients in this group experienced poor outcomes. In contrast, 47.6% had a positive AI signature, of whom 55.6% experienced poor outcomes (109). These findings highlight AI’s ability to classify patients into distinct prognostic groups, particularly those at high risk of poor outcomes.

Similarly, a study on 39 adult B-cell lymphoma patients treated with CD19 CAR-T cells used transfer learning(a deep learning technique) to analyze 770 lymph node lesions. Pre-treatment diagnostic CT (dCT), low-dose CT (lCT) from PET/CT, and FDG-PET images were assessed to predict responses at both lesion and patient levels. Lesion response was defined by size or metabolic activity reduction. Using AlexNet, a pre-trained CNN-MLP, researchers achieved 82% accuracy in lesion-level predictions, outperforming the International Prognostic Index (IPI) in sensitivity, specificity, and accuracy (110).

Beyond imaging-based response prediction, researchers have also applied AI for response prediction using RNA sequencing data. A study of 39 mantle cell lymphoma patient samples (30 sensitive, 9 resistant to CAR-T treatment) employed single-cell RNA sequencing and performed differential gene expression analysis to identify genes that were expressed differently between sensitive and resistant samples. A total of 1,236 different genes were identify. The differentially expressed genes were used as inputs for a MLP. The number of neurons in the input layer corresponds to the number of differentially expressed genes, while the output layer consists of two neurons representing the probability of the patient’s categorical response labels (sensitive or resistant). The model achieved an average accuracy of 90.07% in classifying cells based on the genes expression as sensitive or resistant to therapy (111).

Collectively, these studies highlight AI’s role in improving patient selection and outcome prediction for CAR-T therapy compared to traditional methods and opens new avenues for personalized treatment strategies.

### 4.2 CAR-T cell extraction, isolation and quality assessment

CAR-T cell production is complex and demands strict quality control to ensure safety and efficacy. As the demand for cell and gene therapies increases, automating manufacturing processes will become critical to achieve enhanced standardization. Innovations that eliminate intermediary steps, such as T cell separation using magnetic beads or nanomatrices coated with anti-CD3 and anti-CD28 antibodies (112), can substantially reduce production timelines, resource usage, and contamination risks inherent to manufacturing processes (113). Traditional flow cytometry (FCM) techniques also play an essential role in assessing cell populations; however, conventional methods involving fluorescent staining with antibodies and chemical reagents may compromise cell function, introduce toxicity, and reduce viability (114). Additionally, meeting Good Manufacturing Practice (GMP) standards necessitates specialized personnel, further increasing costs and operational variability (115, 116). These factors hinder the efficient scale-up of CAR-T cell production.

AI-driven sorting systems like ghost cytometry (GC), introduced by K. Sugimoto et al., offer a label-free alternative to antibody-based cell sorting. With this tool developed in 2020, cells are illuminated during imaging to produce distinct optical signatures and waveforms, reflecting various aspects of cell morphology and behavior. ML analyzes these aspects, enabling high-speed sorting when integrated with microfluidics. By training ML classifier with temporal and frequency-domain features extracted from the specific waveforms generated by each cell, allowing the capture of relevant morphological information, GC accurately distinguishes live from dead cells and identifies CD3-positive T cells in peripheral blood mononuclear cells (PBMC) populations (117). By 2024, label-free GC (LF-GC) further improved precision, differentiating live vs. apoptotic cells, T cells vs. non-T cells, and detecting particulate impurities. These advancements enhance real-time cell monitoring and quality control in cell therapy manufacturing without the need for surface markers or stains (118).

Other approaches like COSMOS (Computational Sorting and Mapping of Single Cells), combines high-resolution imaging, DL, and microfluidics for real-time, label-free cell sorting. Using brightfield microscopy, it captures cellular images and analyzes size, shape, and structures. Using deep learning with CNNs and MLPs, it classifies cells by mapping their features into a representation space, enabling accurate and real-time identification (119). Other AI-enhanced systems like DeepCell integrate high-content imaging with neural networks to predict cell identity and function in real time. These platforms enable morphological profiling without staining and support cloud-connected analytics for remote QC, offering potential scalability for GMP-compliant CAR-T production (120).

Computer vision plays a fundamental role in enhancing these image-based sorting technologies by enabling the automated extraction of detailed morphological and structural features from high-resolution cellular images. This field within deep learning allows for accurate, real-time classification without the need for chemical staining, significantly improving throughput, consistency, and viability in CAR-T cell manufacturing. This integration accelerates decision-making and ensures greater standardization in cell quality assessment (121).

### 4.3 CAR design and optimization of T cell function

A key goal is engineering T-cell phenotypes that enhance cytotoxicity while maintaining a stem-like state for long-term persistence, which its traditionally made on trial-and-error methods (110). Neural networks can expedite CAR development by analyzing large datasets that integrate receptor structures with therapeutic outcomes.

One study constructed a library of costimulatory domains by recombining 13 signaling motifs, generating 2,379 unique combinations with distinct phenotypic traits. For instance, the 4-1BB domain enhances persistence, while CD28 boosts cytotoxicity but reduces longevity (122). ML models analyzed these motif interactions and generated diverse T-cell phenotypes, including separate cytotoxicity and stemness traits not typically observed with natural domains. This predictive approach significantly expands exploration of receptor combinations, potentially advancing the development of precise and potent cellular therapies (123).

Costimulatory domains also influence IS quality, directly affecting CAR-T activation and cytotoxicity. Unlike conventional T cells, CAR-T cells form disorganized IS structures, initiating rapid signaling via Lck/ZAP70 and exhibiting “serial killer” activity (17, 55, 56, 124–126). Traditional prediction methods, such as flow cytometry and *in vivo* models, are resource-intensive and inconsistent (127). In response to these challenges, researchers developed a DL tool that improves IS analysis using clinical trial data. They employed a segmentation neural network to accurately delineate kappa-CAR-T cells within the images, followed by a classification network that predicted therapeutic response based on the morphological analysis of the immunological synapse. This approach proved effective even in low-contrast images, accurately distinguishing between treatment responders and non-responders (128). Other approaches use the images obtained by optical diffraction tomography (ODT) combined with DL, which allows automated 3D IS tracking by segmentation and classification, further refining CAR-T assessments (129).

Beyond IS dynamics, tonic signaling is crucial for CAR-T function. This baseline signaling, independent of antigen engagement, must be carefully regulated—too little weakens persistence, while excessive signaling induces exhaustion (130–132). CAR antigen-binding domains with positive charge patches (PCPs) promote clustering, influencing tonic signaling (130). AI-driven tools like CAR-Toner leverage structural data from (Protein Data Bank)PDB and AlphaFold to optimize PCP scores, improving CAR-T expansion and reducing exhaustion. This method was validated in camelid single-domain nanobody (VHH)-based CARs targeting CLL1 in acute myeloid leukemia, identifying an optimal PCP score range (43–52, 255) that correlates with superior CAR-T function (133).

### 4.4 Adverse effect prediction

CAR-T therapies represent a breakthrough in cancer treatment but carry risks such as Cytokine Release Syndrome (CRS), Immune Effector Cell-Associated Neurotoxicity Syndrome (ICANS), and On-Target, Off-Tumor Toxicity (OTOT).These toxicities significantly impact clinical outcomes, requiring effective management strategies and early detection of this complications often rely on clinical observation, scoring and are often subjective and time-intensive (134, 135). CRS, the most common complication, affects 30%–100% of patients, with severe cases (grade ≥3) occurring in 10%–30% (136, 137). It results from excessive cytokine release and endothelial activation, leading to increased vascular permeability, hypoperfusion, coagulopathy, and potential multi-organ dysfunction (138–141).

To enhance early detection, ML models like M2-CRS have been developed. This meta-analysis-informed ML approach integrates statistical data from clinical studies with ten predictive cytokine biomarkers, including IL-2, IL-6, IFN-γ, and GM-CSF. By leveraging a robust Knowledge Base (KB) of cytokine interactions, M2-CRS addresses data scarcity while maintaining high interpretability. The model, based on Support Vector Machines, consistently achieves accuracy and precision above 90%, making it a promising tool for CRS risk stratification and timely intervention. To further enhance interpretability, SHapley Additive exPlanations(SHAP) values were applied, allowing the identification of the most influential features within the model. This analysis revealed IFN-γ and IL-10 as the most relevant proteins contributing to CRS risk prediction in acute myeloid leukemia and B cell Lymphomas (142).

ICANS, another significant CAR-T toxicity, affects 30%–50% of patients, with severe cases in 12%–30% (143). It arises from cerebral endothelial activation, blood-brain barrier disruption, and cytokine-induced astrocyte and pericyte dysfunction, leading to cerebral edema, thrombosis, hemolysis, and disseminated intravascular coagulation (DIC). Logistic regression model can analyze longitudinal patient data faster, predicting ICANS onset and severity with accuracy of 77%. By identifying key risk factors such as age, fever, IL-6 levels, and procalcitonin, ML can forecast ICANS progression up to three days in advance. This predictive capability enhances clinical decision-making, optimizing resource allocation, hospitalization planning, and early therapeutic interventions (144).

## 5 Smart CAR-T Nanosymbionts: AI-nanotech interface empowering living CAR-T therapeutics

The features of nanotechnology and artificial intelligence, previously applied independently in CAR-T therapy, can be seamlessly integrated, offering transformative potential throughout CAR-T design and functionality. Our interface “Smart CAR-T Nanosymbionts” represents an advanced AI-nanotechnology boundary designed to optimize many aspects of CAR-T therapy, including manufacturing, *in vivo* efficacy, and patient monitoring. This model enhances scalability and precision in nanoparticle formulation, facilitates structural modeling and simulations for CAR engineering, and provides real-time monitoring capabilities. Additionally, it proactively detects and manages adverse effects, creating a comprehensive system that maximizes therapeutic performance

### 5.1 AI-Driven optimization of CAR-T cell and nanoparticle manufacturing: from mRNA Delivery to antigen targeting

CAR-T cell and nanoparticle production are interdependent biotechnological processes, requiring precise control for consistency and efficacy. Nanoparticles are essential for mRNA-based CAR expression, facilitating gene delivery to T cells, but large-scale manufacturing faces challenges in maintaining size, charge, and composition. Batch variability affects cellular uptake and gene transfer, making precise formulation critical (145, 146). Similarly, CAR-T cell production demands careful monitoring of viability and expansion in bioreactors.

Integrating real-time monitoring with ML can optimize synthesis parameters, reducing variability and enhancing scalability in both nanoparticle formulation and CAR-T manufacturing (147, 148). Lipid nanoparticles (LNPs) are currently the most widely utilized carriers for mRNA delivery, comprising ionizable lipids, phospholipids, cholesterol, and PEG-lipids (149–151). Ionizable lipids facilitate protein expression, while nanoparticle size and charge critically influence cellular uptake, immune responses, and delivery efficiency; therefore is essential to ensure consistent and effective large-scale production (152–155). XGBoost is a gradient boosting algorithm based on decision trees, known for its high predictive accuracy and computational efficiency in structured data. It builds models iteratively, adding new trees to correct previous errors while balancing accuracy and complexity through regularization. In the context of nanoparticle production, XGBoost has demonstrated significant effectiveness in optimizing nanoparticle production. Sato et al. used XGBoost to predict key parameters in the manufacturing of lipid LNPs, such as particle size, polydispersity index (encapsulation efficiency (EE Independent regression models were built using process variables such as total flow rate, pH, and concentrations of lipids, ethanol, mRNA, and buffer. The models demonstrated high accuracy, with correlation coefficients of 0.998 for particle size, 0.990 for PdI, and 0.977 for EE (156).

Other notable advancement is presented by Amirreza Mottafegh et al. Introducing an autonomous platform for rapidly synthesizing drug-loaded nanoparticles, such as liposomes and polymeric nanoparticles. Their system features an Automated Nanoparticle Synthesizer (ANS) using nanoprecipitation to precisely control flow rates and reactant concentrations. It also incorporates a ML-driven multi-objective Bayesian optimization (MOBO) algorithm, which dynamically adjusts parameters based on real-time feedback. This innovation reduces production time from hours or days to just 20 minutes while ensuring high reproducibility and quality (157). AI-driven optimization in nanoparticle manufacturing can enhance CAR-T therapy by boosting initial production and improving efficiency.

Our proposed AI-revolution could also extend to cell manufacturing with smart bioreactors equipped with nanosensors enabling precise, real-time monitoring and adaptive control of culture conditions. These advanced systems integrate thin-film nanosensors—such as iridium oxide for pH, platinum-based for oxygen, and graphene-coated for glucose—capable of detecting even subtle environmental changes (158–161). For example, a Pd/Pt-functionalized glucose sensor on graphene ensures continuous nutrient monitoring, preventing both starvation and overfeeding. When paired with wireless electronics, these sensors provide continuous feedback, allowing automated adjustments to optimize culture conditions, reduce costs, and enhance scalability without compromising quality (160). AI analytics further enhance this approach by enabling the interpretation of complex, high-dimensional nanosensor data. In the work by Herpin et al., MLP network was employed to disentangle overlapping infrared spectral signals from proteins, lipids, nucleotides, and peptides, enabling real-time monitoring of dynamic molecular interactions with high accuracy (162, 163). Similarly, Leong et al. demonstrated the use of CNNs to analyze multiplexed Surface-Enhanced Raman Scattering (SERS) spectra, achieving over 86.8% classification accuracy in distinguishing small-molecule metabolites such as ATP, glucose, and lactate in complex biological environments. These deep learning models transform intricate biochemical signatures into interpretable and actionable insights, paving the way for real-time diagnostics and precise metabolite monitoring at the point of need (164).

In bioreactors, AI models analyze cell growth, nutrient levels, and environmental fluctuations, dynamically adjusting conditions to optimize expansion. For example, in the work by Grzesik et al. regression models (including Elastic Net and Random Forest) were trained on donor-specific data to predict T cell viability and expansion with high accuracy (R² > 0.92, RMSE < 1.5). These models enabled the in-silico design of optimized culture media, which were experimentally validated and outperformed traditional regression approaches, illustrating how machine learning can streamline media optimization and enhance bioprocess robustness (165, 166).

Beyond manufacturing, it’s important to look at the molecular level to integrate innovative approaches aimed at enhancing CAR-T therapy. Initially, it is key to optimize mRNA delivery for CAR-T therapy success. Within the acidic environment of endosomes, lipid cores become charged, facilitating endosomal escape—a crucial step for successful nucleic acid delivery (167, 168). However, not all nanoparticle formulations perform equally well. Some formulations may accumulate within sub-endosomal membranes, impeding efficient release and consequently reducing transfection efficiency (169). While nanoparticles show great potential, optimizing their performance through conventional trial-and-error methods remains challenging (170).

DL is a powerful tool for overcoming these bottlenecks, enabling the analysis of molecular patterns, deciphering chemical structures, and predicting the behavior of novel substances. Computational models can simulate cationic polymer behavior within endosomes, facilitating the design of more efficient gene delivery systems (171). This significantly reduces the time and cost involved in developing new platforms. AI-driven models also allow detailed study of ion channel physiology and genomics, supporting the design of systems that promote enhanced endosomal and lysosomal escape (96, 172–174). AI can also evaluate critical factors like cargo fusion rates after internalization, optimizing vectors for better performance (26, 175). In this context, multimodal neural networks (which integrate heterogeneous data sources such as chemical structure, physicochemical descriptors, biological response profiles, and imaging data) are emerging as advanced tools for optimizing nanoparticle design. By jointly analyzing multiple modalities, these models offer a more holistic understanding of how structural and functional features interact, allowing for more precise prediction of mRNA transfection outcomes and nanoparticle behavior across complex biological environments (176).

A prominent example of AI’s potential in gene delivery is the AGILE platform, developed by Yue Xu and colleagues. AGILE uses DL, combining combinatorial chemistry with high-throughput screening (HTS), to rapidly discover ionizable lipids optimized for mRNA delivery. At the core of AGILE is a multimodal deep learning model that integrates two complementary inputs: molecular graphs and calculated descriptors. The graph encoder, based on graph neural networks (GNNs), encodes the structural topology of lipid molecules, while a molecular descriptor encoder processes physicochemical features derived from each compound. Initially, the GNN is pre-trained in a self-supervised manner on a vast dataset of small molecules to capture generalizable structural patterns. It is then fine-tuned with experimental data from 1.200 synthesized lipids formulated into lipid nanoparticles (LNPs) and assessed for mRNA transfection potency (mTP) in specific cell lines. This refined, multimodal model predicts the mTP of new lipid candidates, enabling the prioritization and selection of high-performing structures for experimental validation. AGILE identified H9 and R6, ionizable lipids optimized for mRNA delivery to muscle cells and macrophages, respectively. H9 outperformed industry-standard lipids like ALC-0315 (used in Pfizer’s COVID-19 vaccine) in muscle tissue, minimizing liver off-target effects, while R6 exhibited superior transfection efficiency in macrophages (175). By rapidly screening extensive lipid libraries, AI-driven platforms like AGILE can optimize ionizable lipids for T-cell mRNA transfection, improving CAR expression, T-cell expansion, and addressing critical factors for broader clinical adoption.

Optimizing nanoparticle formulations is essential for effective mRNA delivery; however, identifying optimal targets for immune recognition is equally crucial for successful CAR-T therapies. Neoantigens—tumor-specific antigens generated by cancer cell mutations—are fundamental to CAR-T therapy design. Integrating AI into this process can boost neoantigen discovery, significantly improving therapeutic precision and safety. Traditional neoantigen prediction tools primarily assess peptide-MHC binding strengths to identify potential epitopes. While mouse models utilize *in vivo* and *ex vivo* methods to assess immunogenicity, equivalent approaches for humans remain unavailable (177, 178). Additionally, predicted neoantigens often exceed experimentally validated immunogenic peptides, highlighting the need for advanced computational tools capable of accurately predicting neoantigen immunogenicity (179, 180). Here, bioinformatics plays a key role by enabling the integration and interpretation of multi-omic data (such as transcriptomic, proteomic, and genomic profiles) to uncover biologically relevant patterns. When enhanced with AI, especially deep learning, these bioinformatic pipelines become powerful tools capable of modeling complex immune interactions, improving the prediction of truly immunogenic neoantigens and accelerating the design of personalized immunotherapies (181).

A study made by Fabiana Perna et al. demonstrated computational discovery of potential antigens by identifying acute myeloid leukemia (AML) targets—ADGRE2, CCR1, CD70, and LILRB2—via surface proteomics and transcriptomics. These antigens showed high AML cell expression with minimal presence in normal tissues (182). AI-based methods could enhance such processes further by rapidly analyzing data and detecting complex protein expression patterns, significantly improving prediction accuracy and speed (183, 184). ML algorithms like Random Forest and XGBoost effectively predict immunogenic peptide responses. In one study, researchers trained models on a dataset of immunogenic and non-immunogenic peptides, analyzing characteristics like hydrophobicity, peptide size, and amino acid preferences. This allowed the models to predict peptide immunogenicity efficiently, accelerating antigen discovery for immunotherapy (184).

Similarly, the DL-based tool MHCnuggets employs Long Short-Term Memory (LSTM) neural networks to predict peptide binding to MHC class I and II molecules. Its adaptable architecture handles variable-length peptides and integrates both binding affinity measurements and mass spectrometry-derived data, substantially improving prediction performance. The model supports both regression, estimating continuous IC50 binding affinity values, and classification, identifying binders versus non-binders from immunopeptidomic (HLAp) datasets. MHCnuggets rapidly analyzes millions of peptide-MHC interactions, identifying immunogenic mutations across various cancer types. Although CAR-T therapies do not directly rely on MHC molecules, adapting similar approaches could help identify highly specific surface antigens, enriching overall immune responses and enhancing therapy effectiveness (185). Likewise, nanotechnology addresses challenges in neoantigen stability and immunogenicity, improving antigen spreading and delivery. Zhao et al. Showed myeloperoxidase nanovaccines capable of inducing immunogenic cell death (ICD), triggering an immune response cascade, enhancing neoantigen delivery to lymph nodes, and promoting a pro-inflammatory tumor microenvironment. Integrating AI tools like MHCnuggets could optimize this process further by rapidly prioritizing immunogenic neoantigens for personalized cancer immunotherapy (186–188).

It’simportant to mention that, besides the discovery of neoantigens, the quest for highly specific and effective targeting mechanisms leads us to explore alternative binders: Nbs present a promising alternative to traditional scFvs in CAR-T therapy. Due to their smaller size, enhanced stability, reduced immunogenicity, and ability to target unique epitopes, nanobody-based CAR-T cells hold significant potential to improve cancer treatments (76). CAR-Toner has demonstrated that nanobodies-based CARs exhibit optimal positive charge patch (PCP) scores, correlating with improved CAR-T cell functionality (133). Accelerating the discovery of high-affinity nanobodies enables more precise and effective therapeutic interventions. Artificial intelligence further enhances this process, notably through the ML-driven platform NbAffinity, which predicts nanobody-target binding affinity by analyzing critical molecular interactions such as hydrogen bonding, aromatic stacking, and ionic interactions. NbAffinity integrates advanced algorithms—including Random Forest (RF), Rotation Forest (RotF), and Support Vector Machines (SVMs)—to efficiently analyze extensive structural data. Trained on a comprehensive dataset of 991 nanobody-ligand complexes supplemented by 444 protein-protein interaction pairs, NbAffinity achieves robust, generalizable predictions.On the test set, SVM reached an accuracy of 75.87%, reflecting a strong ability to classify affinitive versus non-affinitive nanobodies based on structural interaction features (189).

Building on the precision offered by AI-driven nanobody discovery, it becomes clear that understanding and enhancing dynamic interactions within the tumor microenvironment is equally crucial for advancing CAR-T therapies. Predicting real-time interactions between CAR-T cells, nanoparticles, and tumor cells remains a complex challenge. Emerging technologies like digital twins and virtual patient models are poised to improve cancer treatment by leveraging computational methodologies, including quantitative systems pharmacology (QSP) and physiologically based pharmacokinetics (PBPK), to simulate complex biological processes (190–194). Digital twins provide real-time, personalized tumor replicas, capturing distinct biological and physical attributes. In contrast, virtual patient models incorporate population-level variability, offering valuable insights into diverse subgroup responses (195). These models integrate multi-omics data, predicting tumor dynamics, immune interactions, and therapeutic outcomes, thus enhancing targeting accuracy and minimizing off-target effects (196–198). Such approaches significantly improve simulation of interactions within the CAR-T cell-nanoparticle-tumor axis, optimizing tumor targeting and delivery efficiency (199–202).

Integrating AI further amplifies these capabilities by analyzing tumor morphology, metabolism, and pharmacokinetic profiles (200). This data-driven approach refines tumor microenvironment simulations, optimizing CAR-T cell and nanoparticle design and administration. Platforms such as TumorScopePredict illustrate practical applications by generating dynamic 3D tumor models and accurately predicting therapeutic responses from pre-treatment imaging data processed via CNNs, simulating tumor behavior, drug sensitivity, and resistance (203, 204). The adaptive nature of AI ensures these models remain at the forefront of cancer research, continuously evolving with new data (205, 206).

### 5.2 Precision remodeling of the solid tumor microenvironment: the role of AI-guided nanotechnology in CAR-T cell therapy and vivo manufacturing

One significant challenge for CAR-T therapy in solid tumors is overcoming physical and immunosuppressive barriers within the tumor microenvironment (TME). Unlike hematologic malignancies, solid tumors present dense extracellular matrices (ECM) and aberrant vasculature, impeding T-cell migration. Additionally, immunosuppressive cells such as tumor-associated macrophages (TAMs), regulatory T cells (Tregs), and myeloid-derived suppressor cells (MDSCs) create a hostile environment, limiting T-cell effectiveness through cytokine secretion and checkpoint inhibition (207–213).

Nanotechnology addresses these challenges by remodeling the TME, degrading ECM, and reprogramming immunosuppressive cells like TAMs. It also facilitates targeted delivery of supportive cytokines (e.g., IL-12, IL-15) and immune checkpoint inhibitors (e.g., anti-PD-1) to enhance CAR-T cell performance. Additionally, nanoparticles address antigen escape via multi-antigen targeting and epitope spreading (17, 64, 84, 84, 214, 215). However, clinical translation remains limited due to poor delivery efficiency (DE), with only ~0.67% of nanoparticles reaching the tumor, while the remainder accumulates off-target, primarily in the liver, spleen, and lungs (216–221). This challenge highlights the necessity for improved nanoparticle design to enhance tumor targeting while minimizing systemic distribution.

AI-driven approaches can significantly enhance nanoparticle design, optimizing physicochemical properties to improve tumor targeting and reduce systemic distribution. A DNN utilizing the Nano-Tumor Database (534 tumor datasets, 1972 tissue datasets) accurately predicted nanoparticle DE in tumors and key organs based on nanoparticle properties and therapy strategies. This AI-driven approach identified core materials as critical determinants of DE and provided a web-based tool, Nano-AI-QSAR, facilitating nanoparticle optimization (222). Other organ-specific studies have used AI to optimized brain-targeted nanoparticle delivery, identifying influential factors such as release rate and molecular weight, enhancing nanoparticle effectiveness (223). Additionally, AI-driven optimization of mRNA nanovaccines demonstrated significant improvements in transfection efficiency and lymphatic delivery, successfully activating the stimulator of interferon genes (STING) pathway and boosting antitumor immunity *in vivo* (224). Given the high dimensionality, heterogeneity, and non-linear relationships within biological and physicochemical data, deep learning models are increasingly favored for their capacity to capture complex patterns and interactions that traditional models may overlook (225).

Based on the foundation of AI-optimized nanoparticle design for improved delivery efficiency (DE), an exciting frontier lies in magnetically guided nanoparticles, a strategy ripe for further refinement through AI-driven models. Certain nanoparticles possess magnetic properties that facilitate their targeted redirection to tumor sites—a strategy that can be refined through AI-driven models. Yasmeen Akhtar et al. developed computational simulations integrating factors like magnetic field strength, blood flow, and chemical dynamics to optimize delivery efficiency (DE) and minimize off-target accumulation. Pulsatile flow experiments revealed that stronger magnetic fields enhance nanoparticle retention, while pulsatile conditions improve tumor penetration (62, 226).

Such AI-driven magnetic guidance methodologies hold considerable promise for CAR-T therapy, especially since magnetic control has already demonstrated potential for directing T-cell migration. This suggests substantial opportunities to further apply AI techniques to optimize these processes. Innovative strategies, such as CAR-T-cell-based microrobots (M-CAR Ts), which incorporate immunomagnetic beads onto CAR-T cells, facilitate precise targeting and deep tumor infiltration through sequential magnetic-acoustic actuation, markedly increasing T-cell accumulation and therapeutic efficacy (227). Similarly, CAR-T cells functionalized with superparamagnetic iron oxide nanoparticles (SPIONs) can be magnetically guided to tumor sites, enhancing localized cytolytic activity while simultaneously reducing systemic cytokine release and associated toxicities (228). Integrating AI-driven predictive modeling with advanced nanoparticle engineering can actualize the therapeutic potential of nanotechnology for solid tumors. AI can overcome critical barriers in CAR-T therapy by optimizing nanoparticle properties for superior delivery efficiency, precision targeting, and incorporating complementary approaches such as magnetic guidance to achieve robust and targeted therapeutic outcomes.

Now that the key components for precise and efficient targeted delivery are in place—including AI-optimized nanoparticles and advanced TME-modulating strategies—the prospect of *in vivo* CAR-T cell generation could be within reach. Current CAR T-cell production involves leukapheresis to isolate patient T cells, activation with cytokines and co-stimulatory molecules, genetic modification via lentiviral or γ-retroviral vectors to introduce CAR genes, and subsequent cell expansion into therapeutic doses. This complex and costly procedure requires specialized facilities and trained personnel (14, 229). In contrast, the proposed *in vivo* CAR T-cell production, involving direct in-patient delivery of CAR genes and activation signals, simplifies manufacturing, reduces costs, and enhances accessibility. Preclinical studies in mouse models have shown comparable antitumor efficacy between *in vivo*-produced and *ex vivo*-engineered CAR-T cells (53, 54, 230).

Successful *in vivo* CAR-T cell therapy must meet several critical criteria: high gene-editing efficiency, precise T cell targeting, the ability to overcome solid tumor barriers, sustained functionality and persistence of CAR-T cells, and minimal toxicity (51, 231). CAR T cells face major barriers in the tumor microenvironment (TME), including physical, chemical, and immunological obstacles. To overcome these, nanoparticles are being developed not just for drug delivery but also to modulate the TME and create a “theranostic window” where tumors are both treatable and detectable. These particles (10–200 nm) accumulate in tumors via the EPR effect and can be functionalized with ligands targeting tumor or stromal cells. They can also carry enzymes like collagenase to degrade the ECM, enhancing T cell infiltration and nanoparticle penetration. Additionally, they can deliver siRNA, miRNA, or inhibitors (e.g., TGF-β, IDO, checkpoint blockers) to reprogram the immunosuppressive TME. The Smart CAR-T Nanosymbionts interface exemplifies this advancement, integrating functionalities for precise targeting, enhanced persistence, and minimized toxicity by leveraging the advanced technologies discussed in this paper. Furthermore, AI-driven platforms such as AGILE are being employed to optimize the design of ionizable lipids, significantly enhancing the transfection efficiency of gene delivery systems. This ensures robust CAR expression in T cells, further supporting the development of effective *in vivo* CAR-T therapies (175).

**Figure 4:**
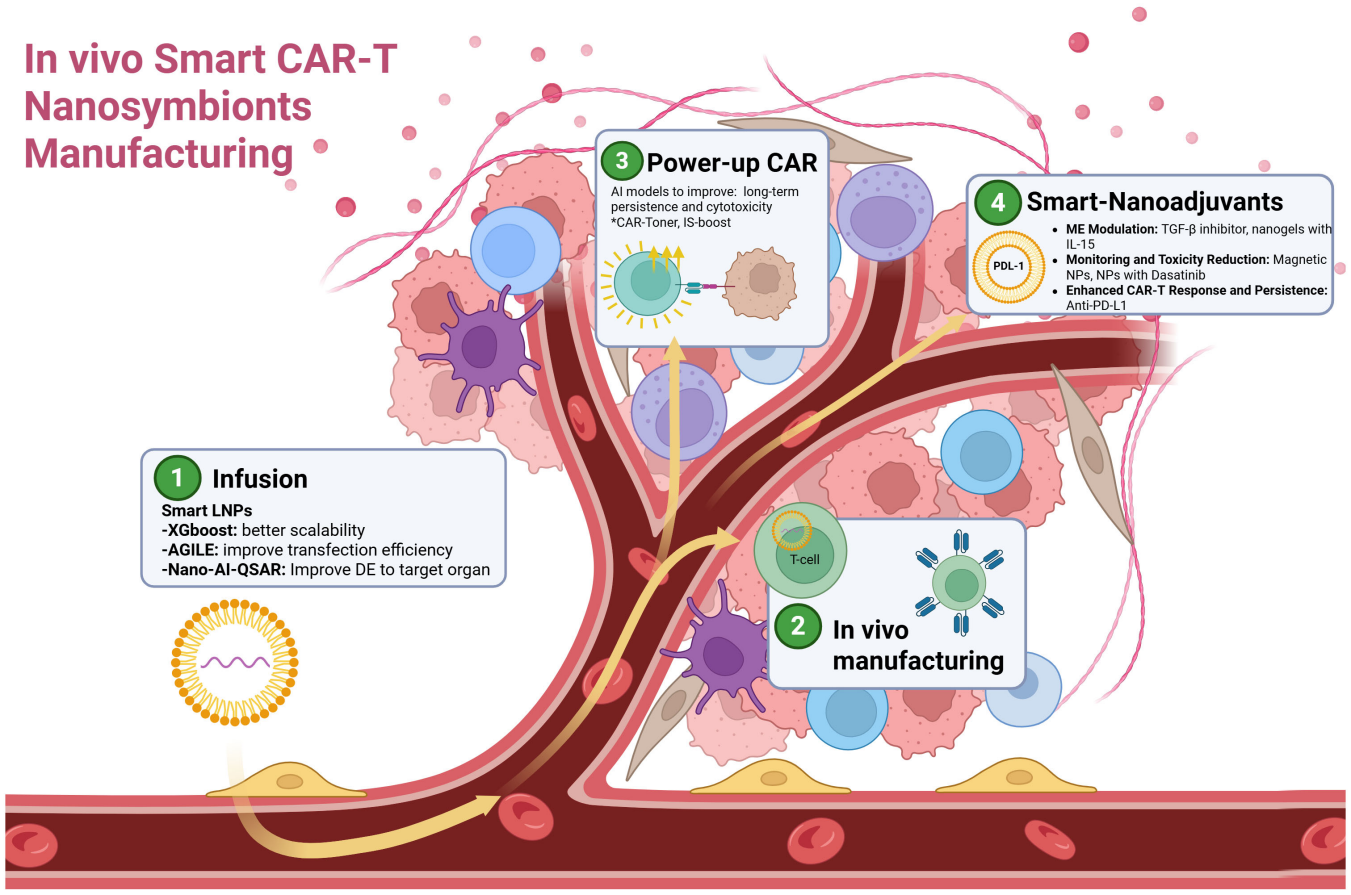
Illustrative schematic of the *In Vivo* Smart CAR-T Manufacturing, highlighting four key phases in targeted cancer therapy: 1. Infusion: Administration of smart lipid nanoparticles optimized with artificial intelligence models such as XGBoost for improved scalability, AGILE for enhanced transfection efficiency, and Nano-AI-QSAR to optimize delivery and gene expression in target organs. 2. *In vivo* manufacturing: Direct conversion of T cells into CAR-T cells within the patient, eliminating the need for *ex vivo* manipulation, reducing production time and costs. 3. CAR-T enhancement: Implementation of AI models to improve CAR-T cell persistence and cytotoxicity through strategies like CAR-Toner to prevent exhaustion, and AI-improved IS + costimulatory domain to enhance cytotoxicity and memory formation. 3. Smart nano-adjuvants: Finally, independent strategies for solid tumors can further be improve with AI(like target delivery) to act in key factors such as; Modulation of the tumor microenvironment using TGF-β inhibitors and IL-15 nanogels, target monitoring and toxicity control with magnetic nanoparticles and Dasatinib, and enhanced CAR-T response and persistence with PD-L1 inhibitors. Created with: Biorender.com. Abbreviations:.

Additionally, AI-guided nanoparticle design improves tumor-specific targeting, minimizing off-target accumulation, facilitating extracellular matrix degradation for improved tumor infiltration, delivering targeted immunomodulatory agents (e.g., IL-12, anti-PD-1), and enabling efficient mRNA delivery into T cells (222). Advanced targeting strategies like magnetically guided M-CAR Ts and SPION-functionalized CAR-T cells enhance tumor localization and deep tissue penetration as necessary (228). Maintaining CAR-T cell functionality involves localized cytokine delivery facilitated by nanotechnology, complemented by AI-driven optimization tools such as CAR-Toner, which refine tonic signaling, and computational approaches optimizing co-stimulatory domains (CD28, 4-1BB) to balance T-cell activation and memory formation directly within the patient (55–60). Finally, addressing potential toxicity necessitates a comprehensive strategy incorporating precise targeting, tumor-specific neoantigen prediction, and controlled-release systems for cytokines and checkpoint inhibitors, collectively ensuring both safety and therapeutic efficacy (232–234). **Figure 4** represents the congregation of these technologies in the “*In vivo* Smart CAR-T Nanosymbionts manufacturing”.

### 5.3 Clinical applications of AI-enhanced monitoring of CAR-T: imaging, tracking, and toxicity detection

AI-driven image analysis has the potential to revolutionize cancer imaging by shifting from qualitative interpretation to objective, quantitative evaluations. This transformation facilitates earlier detection, precise lesion characterization, and improved monitoring of disease progression and treatment responses (235). In CAR-T therapy specifically, multimodal network models integrating imaging and clinical data have successfully predicted treatment outcomes (109, 110).

Iron and gold nanoparticles have emerged as powerful contrast agents for magnetic resonance (MR), computed tomography (CT), and positron emission tomography (PET) imaging, significantly enhancing the resolution and visibility of both solid tumors and hematological malignancies. These advancements not only improve diagnostic accuracy but also reduce imaging duration and resource utilization (236–239). When combined with AI-driven innovations in biodistribution and targeted delivery, nanoparticles further amplify tumor contrast, enabling precise lesion delineation and superior assessment of therapeutic responses. CNNs trained on nanoparticle-enhanced imaging data refine tumor characterization and support real-time clinical decision-making, fostering automated and precise cancer monitoring (240). Additionally, *in vivo* tracking of CAR-T cell distribution with contrast agents enhances persistence and tumor targeting. This enables real-time response assessment and better lesion characterization (228). The previously mentioned CNN-based approach could further improve non-invasive CAR-T tracking methods, such as ferumoxytol-enhanced MRI for iron oxide- or gold-labeled CAR-T cells, optimizing the evaluation of therapy distribution and effectiveness (20, 21, 88).

Besides imaging monitoring, tracking CAR-T cells in peripheral blood is critical for assessing therapy efficacy and ensuring patient safety, particularly in blood malignancies like acute leukemia (241). Traditional identification methods are limited due to the similarities between CAR-T cells and other immune cells. However, the RCMNet model, which integrates CNN-MLP trained on Peripheral Blood Cells (PBC) datasets, achieves a top-1 accuracy of 99.63%, revolutionizing CAR-T cell identification in blood samples. This approach alleviates manual evaluation burdens, provides real-time automated analysis, and markedly improves diagnostic accuracy (242). Transformers, originally developed for natural language processing, rely on self-attention mechanisms that allow the model to weigh the importance of different input features relative to one another (243). In this context, they enhance the model’s ability to focus on subtle, context-dependent cellular traits that differentiate CAR-T cells from other immune populations.

While monitoring therapeutic responses is crucial, it is equally vital to detect adverse effects such as cytokine release syndrome (CRS) and immune effector cell-associated neurotoxicity syndrome (ICANS) at an early stage. Nanoelectronics biosensors, such as silicon nanowire field-effect transistors (SiNW FETs), provide highly sensitive, real-time detection capabilities that surpass traditional methods like ELISA (244, 245). These nanosensors enable precise tracking of cytokines and toxicity markers, facilitating rapid clinical intervention—all of which can be further optimized and enhanced through AI-driven models (246). The detailed data generated by these biosensors can strengthen AI-driven predictive models, such as M2-CRS, facilitating early toxicity predictions and personalized treatment management. Additionally, machine learning algorithms, particularly Random Forest (RF), can integrate nanoparticle physicochemical properties—such as size, shape, surface coating, and zeta potential—along with exposure conditions like dose, duration, and tissue type to accurately predict nanoparticle-induced toxicity. Such predictive models improve safety assessments, enabling proactive risk management and tailored therapeutic adjustments (247).

## 6 Technological convergence in CAR-T therapies: challenges, limitations, and future directions

The convergence of CAR-T therapy with AI and nanotechnology marks a turning point in personalized oncology. These technologies offer complementary advantages: AI enables data-driven prediction and adaptation, while nanotechnology facilitates controlled delivery and improved CAR-T functionality. Yet, this synergy introduces multilayered challenges that span scientific, ethical, and regulatory domains.

At the scientific frontier, multiple ML and DL models have been integrated across CAR-T development stages, from receptor design to toxicity prediction. CNNs support high-resolution classification and three-dimensional immunological synapse analysis (119, 129); support vector machines (e.g., M2-CRS) can predict cytokine release syndrome (142); MLPs aid signal separation (162); and hybrid models like RCMNet blend CNNs with transformers to identify CAR-T cells in blood samples (242). Platforms such as AGILE combine GNNs and MLPs for lipid discovery in mRNA transfection (175), while LSTM models like MHCnuggets predict peptide–MHC binding (185). However, none of these approaches have yet reached a level of technological maturity suitable for commercial implementation (TRL ≤ 5), and at the time of writing this paper, there is no clinical trials are on the way. A summary of ML/DL models applied to CAR-T therapy can be found at **Table 4**.

**Table 4:** Summary of ML/DL models applied to CAR-T therapy: applications, and TRL.

Model Type	Application	TRL	Reference
ML	Patient selection and response prediction (FDG PET/CT in lymphoma)	5	(109)
ML - SVM	M2-CRS model for predicting cytokine release syndrome	4	(142)
ML - XGBoost	LNP optimization for size, PdI, and EE	4	(157)
ML & DL - Elastic Net and Random Forest	T cell viability	5	(165, 166)
ML - Random Forest/RotF/SVM	Affinity prediction in nanobodies (NbAffinity)	3	(189)
DL - AlexNet	PET/CT image analysis for response prediction in lymphoma	4	(110)
DL - CNN	Cell classification and 3D analysis of the immunological synapse	4	(119, 129)
DL – Segmentation + Classification	Immunological synapse analysis	3	(128)
DL - CAR-Toner	PCP optimization in CARs (structure-function)	3	(133)
DL - MLP	Cellular signal separation	4	(162)
DL - AGILE (GNN + MLP)	Ionizable lipid discovery for mRNA transfection	4	(175)
DL - LSTM (MHCnuggets)	Peptide-MHC binding prediction	4	(185)
DL - RCMNet (CNN + Transformer)	Identification of CAR-T cells in peripheral blood	4	(242)
DL	Classification of sensitive or resistant cells using transcriptomics	4	(111)
ML – Logistic Regression	ICANS prediction	3	(144)
ML - Random Forest, XGBoost	Peptide immunogenicity prediction	3	(184)

Technology Readiness Levels (TRL) are a type of measurement system used to assess the maturity level of a particular technology.

**1–3:** Basic research or proof of concept.

**4–6:** Validation in laboratory or simulated environment.

**7–9:** Implementation in real-world or commercial environment.

A central concern is data integrity. Biased or incomplete datasets can skew AI outputs, introducing disparities in therapeutic prediction or patient stratification. Algorithmic bias—driven by unbalanced training data or non-generalizable architectures—threatens equity across population subgroups (248). Moreover, the probabilistic nature of AI decisions challenges established paradigms of clinical accountability and informed consent. Interpretability tools have emerged to audit these models (93), and explainable AI is gaining regulatory traction (249), yet the gap between computational abstraction and clinical utility remains.

To improve system robustness, data preprocessing, outlier detection via unsupervised learning, and synthetic data generation (e.g., augmentation) are actively explored. Still, generalization in low-data settings remains a core limitation. This demands interoperable clinical databases and the use of synthetic sampling or generative AI to enrich training sets (250). Standardization of model documentation and traceability is essential to harmonize with emerging governance standards and enable consistent model retraining in non-stationary biological environments.

At the nanoscale, delivery systems unlock new routes for CAR-T engineering. Nanoparticle platforms enable the potential for *in vivo* generation of CAR-T cells, bypassing the need for centralized manufacturing. However, unresolved concerns around long-term biocompatibility, off-target immune effects, and pharmacokinetic variability persist. Efficiency of delivery to solid tumors remains a major bottleneck, necessitating novel chemistries, adaptive functionalization, and ligand-directed targeting to increase specificity while minimizing systemic exposure (251).

The fusion of these technologies disrupts traditional regulatory classification. CAR-T therapies enhanced by nanodevices and AI systems defy existing frameworks, which were not designed to evaluate dynamic, self-learning systems or hybrid constructs that combine genetically modified cells, synthetic vectors, and algorithmic decision layers. While they may theoretically qualify as combination products, the lack of harmonized guidance across nanoparticle characterization, AI validation, and cell-processing protocols hinders regulatory review. Existing pathways cannot adjudicate how these components interact to affect dosing, targeting, and real-time decision-making.

Compounding this, there is a dearth of standardized protocols for validating AI-governed nanoparticle formulations under physiological stress, particularly in immunologically diverse populations. The absence of nano-toxicological assays capable of modeling long-term safety profiles further constrains clinical translation. In this context, regulatory frameworks established for mRNA vaccines—already approved and deployed in humans—could serve as a foundational reference to streamline the evaluation of lipid-based delivery systems and accelerate safe clinical adoption. Without robust retraining strategies, AI systems are prone to model drift and loss of predictive performance/failure modes that could compromise patient safety in real-world settings.

Ethical and translational risks are also something to address. As algorithms increasingly guide critical interventions, from patient selection to adaptive dosing, they raise concerns about equity, transparency, and liability. The opacity of high-dimensional models challenges informed consent, while layered technological mediation complicates assignment of clinical responsibility. Regulatory bodies currently lack the tools to evaluate how these algorithmic and biological elements co-evolve, necessitating novel oversight structures that ensure longitudinal monitoring, auditability, and preservation of clinician agency.

Moving forward, three strategic priorities must be addressed to realize the promise of Smart CAR-T Nanosymbionts:

First, AI tools must be developed for generalization in data-scarce, heterogeneous clinical environments. Centralized, standardized repositories and controlled access to interoperable datasets are foundational. Data augmentation techniques and generative models can mitigate sampling limitations (250), but interpretability remains vital. All outputs must be benchmarked against clinical criteria to ensure trust and biomedical validity (252).

Second, nanoparticle platforms require enhanced specificity and safety. Efficient tumor targeting, high-fidelity gene transfection, and reduced immunogenicity are prerequisites for *in vivo* CAR-T generation. These aspects require mechanistic insight into how nanoparticle properties influence cell activation, exhaustion, and biodistribution. Standardized assays correlating particle chemistry with toxicity and organ accumulation are essential.

Third, interdisciplinary integration is paramount. Teams spanning AI, nanomedicine, immunology, and clinical oncology must co-develop scalable protocols and shared data infrastructures. Regulatory harmonization should evolve in parallel to support transparency, patient safety, and equitable access as these technologies transition to clinical settings (253, 254).

In summary, the integration of AI and nanotechnology into CAR-T therapy represents a technological opportunity. Despite this, only through ethical, scientifically rigorous, and regulatory-aware frameworks can these multilayered platforms move from conceptual models to safe, effective, and accesible next-generation therapies.

## 7 Conclusions

CAR-T therapy has revolutionized modern oncology, but its large-scale application faces significant challenges, including complex manufacturing processes, high costs, limited efficacy in solid tumors, and potentially severe toxicities. Emerging technologies such as nanotechnology and AI offer innovative tools to address these limitations, greatly enhancing therapeutic precision and scalability. While both have demonstrated individual promise, their full potential can only be realized through strategic and synergistic integration. The Smart CAR-T Nanosymbionts framework represents a paradigm shift in this direction. It is designed to converge the capabilities of AI and nanotechnology into a unified therapeutic interface that enhances every stage of CAR-T therapy, from design and engineering of CAR constructs to their delivery, *in vivo* behavior, and monitoring. By incorporating AI-driven modeling and optimization, this approach enables dynamic adjustments to manufacturing conditions, personalized antigen targeting, and real-time monitoring of patient-specific responses. Simultaneously, the use of nanotechnology allows for non-viral gene delivery, tumor-specific modulation of the microenvironment, and spatially controlled release of cytokines and inhibitors that enhance CAR-T cell persistence and reduce immune-related toxicities. Moreover, the Smart CAR-T Nanosymbionts challenge boundaries by introducing the possibility of *in vivo* CAR-T generation. This vision leverages AI-optimized lipid nanoparticles to deliver CAR constructs directly into patient T cells, bypassing the need for *ex vivo* manipulation. Such a system could radically simplify production, reduce timelines and costs, and expand access to cell-based therapies, particularly in low-resource settings.

**Figure 5:**
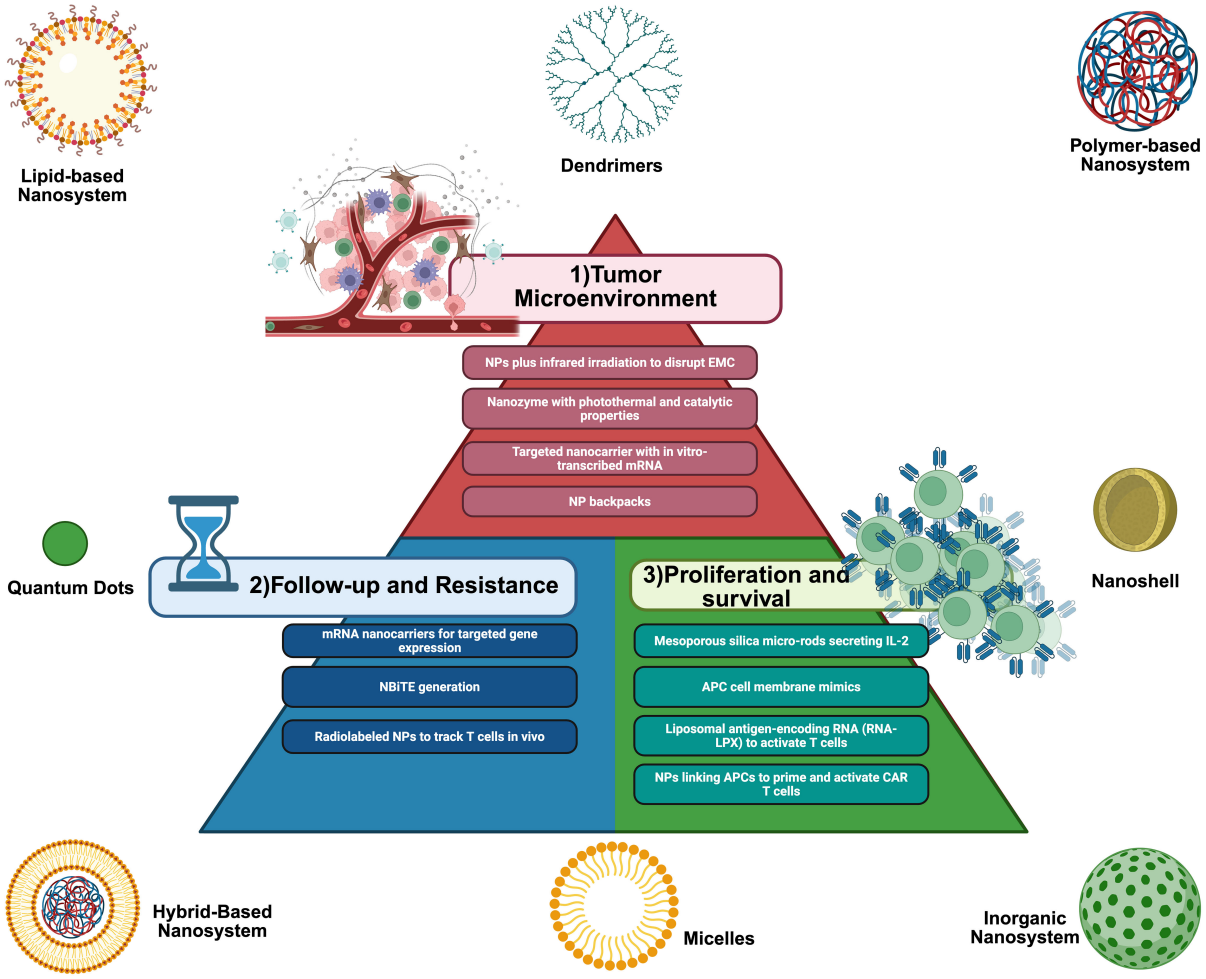
Graphical Abstract of Addition by Subtraction: Smart CART Nanosymbionts: AI serves as the central integrative engine, leveraging machine learning and deep learning algorithms to drive decision-making, pattern recognition, and predictive modeling. This AI-driven framework enhances the design, function and specificity of nanoparticles—such as lipid and -based nanoparticles—and improves CAR-T cell therapy by optimizing patient selection, target specificity, response prediction, and toxicity control. Together, these elements enable the development of intelligent, adaptive, and personalized cancer treatments.

As a complementary conceptual tool, our group has previously developed the Addition by Subtraction model, an operational philosophy aimed at amplifying therapeutic outcomes by systematically eliminating inefficiencies, redundancies, and sources of toxicity across biological and technological layers (17). Although originally proposed in a different context, this model aligns with the foundational goals of Smart CAR-T Nanosymbionts and serves as a guiding principle for integrating innovation in a way that is both scalable and clinically actionable (**Figure 5**; **Table 5**).

**Table 5:** Comparison between conventional CAR-T therapy and smart CAR-T nanosymbionts.

Feature	Conventional CAR-T Therapy	Smart CAR-T Nanosymbionts (Proposed)
Gene delivery vector	Lentiviral/retroviral	AI-optimized lipid nanoparticles (e.g., AGILE, XGBoost) for better transfection efficiency
Manufacturing method	*Ex vivo* in GMP-certified facilities	Potentially: *In vivo* assisted by nanoparticles and predictive algorithms for tumor localization
Production time	14–21 days	No clinical studies have been made to validate time reduction with this approach, although, again Therapy initiation time would benefit from *in vivo* strategies, and higher scalability of nanoparticles and automating platforms.
Average cost per patient	$400,000–$1,000,000 USD	Automating platforms can reduce the cost of therapy by 50%. Nanoparticles would support this model by having higher scalability and lower cost than viral vectors (255–258)
Persistence and durability	Variable, risk of exhaustion	Improved with IL-15 nanogels, controlled-release platforms, AI-optimized stimulation domains boosting cytotoxicity and improve longevity
Efficacy in solid tumors	Poor, limited by tumor microenvironment (TME)	TME remodeling with nanoadjuvants, AI-enhanced targeting, magnetic guidance
Patient selection strategy	Based on clinical and lab criteria	Multimodal AI analysis (e.g., imaging, transcriptomics, CNN, MLP models) with higher response-prediction that standard models
Antigen targeting	Common tumor antigens (e.g., CD19, BCMA)	AI-guided neoantigen discovery (e.g., MHCnuggets, NbAffinity)
Gene expression profile	Permanent (viral integration)	Temporary, non-integrative (mRNA via LNPs): enabling transient, non-integrative gene expression. This allows for multiple infusions to dynamically adjust gene expression as needed throughout treatment.
Toxicity profile	High incidence of CRS/ICANS (30–70%)	Active prevention via AI-guided prediction and nanoparticle-mediated modulation (e.g., Dasatinib, PD-L1 inhibitors)
Regulatory challenges	Known, already with FDA and other internation approval.	Uncharted combination of AI–nano–cell therapy; regulatory precedents lacking Emerging therapy without a standard regulatory process.

Ultimately, the integration of AI, nanotechnology, and CAR-T therapy holds the promise of transforming cancer care by enabling truly adaptive, intelligent, and personalized treatments. However, realizing this vision requires not only scientific advancement, but also regulatory evolution, ethical vigilance, and interdisciplinary collaboration. Future research must prioritize the development of robust translational pipelines, real-time monitoring systems, and equitable frameworks to ensure that these next-generation therapies are safe, accessible, and impactful across global health systems.

To clearly illustrate the transformative potential of this integrated approach, we present a comparative framework between conventional CAR-T therapy and the Smart CAR-T Nanosymbionts model. This side-by-side analysis highlights how the convergence of artificial intelligence and nanotechnology can address critical limitations in manufacturing, safety, cost, and clinical scalability.
